# Adjuvant composition and delivery route shape immune response quality and protective efficacy of a recombinant vaccine for *Entamoeba histolytica*

**DOI:** 10.1038/s41541-018-0060-x

**Published:** 2018-06-05

**Authors:** Mayuresh M. Abhyankar, Mark T. Orr, Susan Lin, Mohammed O. Suraju, Adrian Simpson, Molly Blust, Tiep Pham, Jeffrey A. Guderian, Mark A. Tomai, James Elvecrog, Karl Pedersen, William A. Petri, Christopher B. Fox

**Affiliations:** 1 Division of Infectious Diseases and International Health, Department of Medicine, University of Virginia Health System, Charlottesville, VA USA; 2 IDRI, 1616 Eastlake Ave E, Seattle, WA USA; 3 Department of Global Health, University of Washington, Seattle, WA USA; 4 3M Drug Delivery Systems, 3M Center, 275-3E-10, St. Paul, MN USA; 5 TECHLAB, Inc., 2001 Kraft Drive, Blacksburg, VA USA

## Abstract

Amebiasis caused by *Entamoeba histolytic*a is the third leading cause of parasitic mortality globally, with some 100,000 deaths annually, primarily among young children. Protective immunity to amebiasis is associated with fecal IgA and IFN-γ in humans; however, no vaccine exists. We have previously identified recombinant LecA as a potential protective vaccine antigen. Here we describe the development of a stable, manufacturable PEGylated liposomal adjuvant formulation containing two synthetic Toll-like receptor (TLR) ligands: GLA (TLR4) and 3M-052 (TLR7/8). The liposomes stimulated production of monocyte/macrophage chemoattractants MCP-1 and Mip-1β, and Th1-associated cytokines IL-12p70 and IFN-γ from human whole blood dependent on TLR ligand composition and dose. The liposomes also demonstrated acceptable physicochemical compatibility with the recombinant LecA antigen. Whereas mice immunized with LecA and GLA-liposomes demonstrated enhanced antigen-specific fecal IgA titers, mice immunized with LecA and 3M-052-liposomes showed a stronger Th1 immune profile. Liposomes containing GLA and 3M-052 together elicited both LecA-specific fecal IgA and Th1 immune responses. Furthermore, the quality of the immune response could be modulated with modifications to the liposomal formulation based on PEG length. Compared to subcutaneous administration, the optimized liposome adjuvant composition with LecA antigen administered intranasally resulted in significantly enhanced fecal IgA, serum IgG2a, as well as systemic IFN-γ and IL-17A levels in mice. The optimized intranasal regimen provided greater than 80% protection from disease as measured by parasite antigen in the colon. This work demonstrates the physicochemical and immunological characterization of an optimized mucosal adjuvant system containing a combination of TLR ligands with complementary activities and illustrates the importance of adjuvant composition and route of delivery to enhance a multifaceted and protective immune response to amebiasis.

## Parasitic diarrhea: formulating an optimal vaccine booster

A vaccine formulation optimized with efficacy-boosting constituents offers a protective response against a refractory pathogen. *Entamoeba histolytica* is a leading cause of global mortality, causing diarrheal disease and posing a particular danger to infants. Building on their previous research, a team of US scientists led by the Infectious Disease Research Institute’s Christopher Fox developed a formulation for co-administration alongside their vaccine candidate—an adjuvant—that boosted the efficacy of the vaccine and allowed them to simplify the treatment regimen to three intranasal administrations. During tests, the intranasal vaccine and adjuvant combination provided a preferable immune response compared to vaccination via injection. In experiments on mice, the team’s treatment protocol enhanced protection against *E. histolytica*. This research highlights the importance of route of administration and vaccine composition to vaccine efficacy.

## Introduction

Amebiasis, a diarrheal disease caused by the enteric protozoan parasite *Entamoeba histolytica* (a biodefense category B pathogen), is responsible for ~50 million infections annually worldwide, causes traveler’s diarrhea, and disproportionately affects disadvantaged populations.^1,2^ For example, cases of amebiasis disease in Mexico from 2000–2010 dramatically outnumbered the combined number of cases of dengue, tuberculosis, malaria, AIDS, leishmaniasis, Chagas disease, and multiple other infectious diseases.^3^ Moreover, the risk of death for infected young children is among the highest of all diarrheal diseases as determined in the Global Enteric Multicenter Study.^4^ Although there is currently no vaccine available or in clinical testing, it is known that human immunity is associated with fecal IgA directed against amebic surface lectin and IFN-γ production from peripheral blood mononuclear cells in response to amebic lysate. Preschool children from a Bangladesh cohort showing *E. histolytica*-specific gut IgA and systemic IFN-γ response were protected from recurrent infections.^5,6^

*E. histolytica* infection starts with the ingestion of amebic cysts from contaminated food or water, which after excystation form trophozoites in the intestinal lumen. These adhere to epithelial cells through interaction of galactose and N-acetyl-D-galactosamine (Gal/GalNAc)-specific lectin with host Gal/GalNAc-containing glycoconjugates. The trophozoites kill and ingest host cells in a lectin-dependent manner.^7,8^ Blockade of lectin activity with Gal or GalNAc prevents contact-dependent cytolysis.^8^ Similarly, monoclonal antibodies against the 170-kDa lectin heavy-chain subunit (hgl) completely eliminate the galactose-specific adherence of *E. histolytica* trophozoites to colonic mucins in vitro.^9^ Vaccination with either parasite-purified native lectin or recombinant lectin subunits containing parts of cysteine-rich extracellular domain of the heavy chain combined with potent water-in-oil emulsion or cholera toxin adjuvants provides protection against amebiasis.^10–14^ Important for vaccine development, the sequence of the *hgl* genes is nearly completely conserved in isolates of *E. histolytica* from different continents.^15^

We have developed and manufactured recombinant subunit vaccine “LecA” (amino acids 578–1154 of hgl).^16^ It is a major target of cell-mediated and humoral response of immune individuals, its sequence is conserved in every *E. histolytica* isolate ever examined, and it contains all of the antibody neutralizing epitopes on the molecule.^12,15,17^ However, as a highly pure recombinant antigen, it is poorly immunogenic without the aid of adjuvant technology. Since both IgA and IFN-γ are associated with amebiasis immunity in humans, we sought to develop an adjuvant formulation amenable to parenteral and mucosal delivery with the potential to enhance both mucosal antibody as well as Th1 cell responses. Activation of multiple TLRs more closely represents the mechanisms activated by effective live-attenuated vaccines.^18^ TLR4 and TLR7/8 ligand combinations have shown particular promise in strengthening Th1 immunity and inducing long-lived neutralizing antibodies.^19–21^ In previous work, we screened various adjuvant formulations for their ability to generate a Th1-indicating IgG2a/IgG1 ratio in mice after subcutaneous administration with LecA, thus identifying a PEGylated liposomal formulation containing a combination of synthetic TLR4 (GLA) and TLR7/8 (3M-052) ligands as a promising candidate.^22^ Further evaluation demonstrated that this adjuvant formulation upregulated production of IFN-γ and IL-17A,^22^ cytokines shown to be protective in a mouse model, after subcutaneous administration with adjuvanted LecA.^11,23^ When administered via alternating intranasal and subcutaneous routes with LecA, the GLA-3M-052 liposome formulation generated functional fecal IgA and 34% protective efficacy as assessed by culturing of parasites from cecal contents in a mouse challenge model of amebic colitis.^22^

In the present work, we have focused on evaluating the physicochemical stability and immunological effects of liposome composition and immunization regimen, with the goal of optimizing adjuvant potency. Importantly, these studies demonstrate that a comprehensive mucosal and systemic immune response necessary for protection against amebic colitis can be achieved by a simplified intranasal-only immunization regimen of the adjuvanted vaccine. Furthermore, complementary roles for the dual TLR ligands and the surprising impact of excipient structure on adjuvant biological activity and stability are elucidated. Together, these findings have resulted in an optimized vaccine composition suitable for advanced development as an amebiasis vaccine candidate. Moreover, the adjuvant formulation developed could be applicable for other enteric diseases requiring mucosal antibodies and a Th1-type immune response profile.

## Results

### Liposome compositions

The liposome formulation developed in our previous work consisted of dipalmitoyl phosphatidylcholine (DPPC), PEGylated dipalmitoyl phosphatidylethanolamine with PEG MW of 750 (DPPE-PEG750), and cholesterol.^22^ To determine the roles of GLA and 3M-052, and whether both TLR ligands were necessary for optimal adjuvant activity, we generated liposomes containing GLA or 3M-052, or both. To determine the effect of PEG length on stability and adjuvant activity, we manufactured liposomes containing the same molar concentration of PEG MW 750 or MW 2000. We also evaluated the effects of different TLR ligand concentrations on liposome stability. We note here that, due to reagent availability considerations, it was necessary to switch from the dipalmitoyl PEGylated lipid acyl configuration (DPPE-PEG) used in our previous work to distearoyl (DSPE-PEG) in the present work, unless noted otherwise.

### Physicochemical stability

**Fig. 1:**
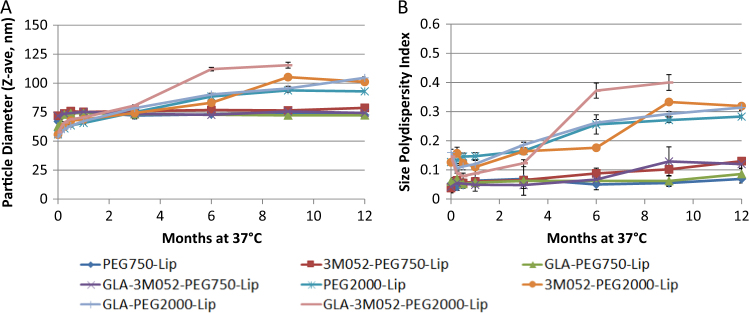
Effect of PEG length and adjuvant composition on liposome physical stability. **a** Particle size and **b** size polydispersity of PEGylated liposomes with indicated compositions and stored at 37 °C. Error bars represent the standard deviation from nine measurements (three measurements from each of three cuvettes)

Liposome stability was monitored by dynamic light scattering (particle size and size polydispersity), visual appearance, and reverse-phase HPLC (GLA and 3M-052 concentrations). The size polydisperstity index is a dimensionless number that indicates the width of the particle size distribution, with values below 0.2 indicating a mostly monodisperse distribution. The initial particle size of liposomes containing PEG2000 was somewhat smaller than liposomes containing PEG750, whereas the size polydispersity values for PEG2000 liposomes were higher than for PEG750 liposomes (Fig. S1). Nevertheless, particle size and size polydispersity values for PEG2000 and PEG750 liposomes showed little change over 12 months when stored at 5 °C (Fig. S1). Likewise, the visual appearance of the liposomes was consistently translucent and homogeneous and did not change over time when stored at 5 °C. When stored at 37 °C, liposomes containing PEG2000 showed greater change in particle size and size polydispersity over time compared to liposomes containing PEG750, which remained remarkably stable (Fig. 1). Increasing the TLR ligand concentrations by 5-fold appeared to result in slightly less stable PEG750 liposomes for some compositions when stored at 37 °C, whereas no difference was apparent with PEG2000 liposomes (Fig. S2). Overall, the presence of one or both TLR ligands did not have detrimental effects on the physical stability of the liposomes when stored for 12 months at 5 °C although a slightly higher polydispersity value was noted for the PEG750 liposome containing high concentrations of both TLR ligands (Fig. S2). Although lower size polydispersity values are generally preferred, the differences in polydispersity values reported here would not be expected to significantly impact manufacturability of the liposome compositions described.

Formulations were analyzed by HPLC-CAD for GLA content after manufacture and the GLA concentration was within 20% of the target value for all batches. After 12 months of storage at 5 °C, GLA content was generally stable and still within 20% of the target value (Fig. S3). Regarding 3M-052 content, six out of eight batches were within the target concentration after manufacture, whereas two batches showed low 3M-052 content, indicating that not all of the 3M-052 was recovered during the manufacturing process in these batches. After 12–16 months storage at 5 °C, the 3M-052 content for all batches was within 20% of the initially measured values (Fig. S3), although the data should be interpreted with caution since different HPLC methods were employed (see Methods section).

To further investigate the chemical stability of GLA and 3M-052, we subjected the GLA-3M-052-PEG2000 liposome batch containing 0.5 mg/ml GLA and 0.2 mg/ml 3M-052 to storage at elevated temperatures (ambient, 37 or 60 °C). This batch had been stored for approximately 12 months at 5 °C prior to this study. When stored at ambient temperature for 8 weeks, no change in GLA or 3M-052 content was apparent (Fig. S3). At 37 and 60 °C, the rate of GLA loss appeared to be somewhat greater than that of 3M-052, indicating that GLA may be more heat labile than 3M-052 in this formulation, although loss of some GLA content would not necessarily be reflected in mouse immunogenicity readouts.^24^ Overall physicochemical stability data indicate that GLA-3M-052 liposomes could be a candidate for a controlled temperature chain scenario (i.e., stable for limited time outside of 2–8 °C conditions).

### Compatibility with LecA antigen

Since the adjuvant is designed to be mixed with the antigen immediately prior to immunization, it is important to establish the short-term (e.g., up to 24 h) physicochemical compatibility of the antigen-adjuvant mixture. To evaluate the short-term (≤24 h) compatibility of adjuvant and LecA antigen after mixing, stock antigen was diluted in saline and then mixed in 1:1 volume ratio with adjuvant containing target concentrations of 0.1 mg/ml GLA and 0.04 mg/ml 3M-052 to mimic the planned mixing procedure for the in vivo subcutaneous immunization studies described below (actual measured concentrations ranged from 0.088 to 0.118 mg/ml for GLA and 0.027 to 0.039 mg/ml for 3M-052). Formulations had a translucent, homogeneous appearance before and after mixing with antigen. Overall, there was little (<15%) or no change in particle size over 24 h after mixing with antigen whether stored at 5 °C or ambient temperature (Fig. S4), although liposomes containing DSPE-PEG2000 showed a slightly greater tendency to increase in size compared to the liposomes containing the shorter PEG length (DSPE-PEG750). Overall, the presence of GLA, 3M-052, or both agonists together did not appear to affect the particle size compatibility results. Likewise, size polydispersity values changed little (Fig. S4). SDS-PAGE analysis of the antigen-adjuvant mixtures indicated no change in antigen primary structure at any timepoint or storage condition over 24 h, regardless of liposome composition, with a uniform single band near the expected MW (Fig. S4).^16^ When component concentrations were increased to support the intranasal immunization composition (resulting in target concentrations of 0.25 mg/ml LecA, 0.25 mg/ml GLA, and 0.1 mg/ml 3M-052 after mixing), liposome particle size increased immediately after mixing with LecA to >30% the size of the adjuvant before mixing (67–87 nm), with only small increase in size thereafter (18 h after mixing the size was 95 nm); moreover, the SDS-PAGE profile of LecA remained unchanged in the presence of the adjuvant (data not shown). Overall, these data indicate that the antigen-adjuvant mixture demonstrated acceptable short-term compatibility for up to 24 h at 5 °C or ambient temperature, thus supporting a “bedside mix” approach immediately prior to immunization.

### In vitro biological activity

**Fig. 2:**
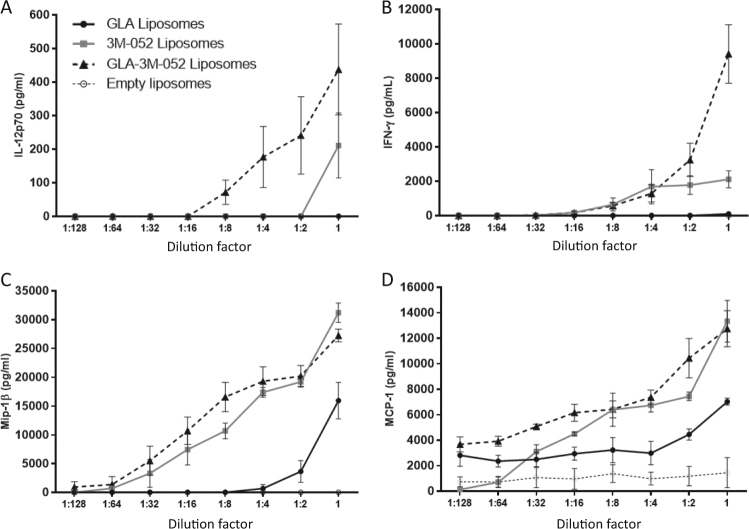
Cytokine production from human whole blood stimulated by liposomes containing GLA, 3M-052, both TLR ligands, or neither (empty). Stimulated whole blood was analyzed for production of Th1-associated cytokines **a** IL-12p70 and **b** IFN-γ, as well as chemokines **c** Mip-1β and **d** MCP-1. The *x*-axes represent serial dilutions with starting concentrations of 4 µg/ml for GLA and 1.5 µg/ml 3M-052. The *y*-axes represent concentrations of target analytes secreted post whole blood stimulation with error bars representing the standard error of the mean from three blood donors with each individual value averaged from duplicate wells

We evaluated production of the chemokines monocyte chemoattractant protein 1 (MCP-1) and macrophage inflammatory protein 1-beta (Mip-1β), and Th1-associated cytokines IL-12p70 and IFN-γ from human whole blood stimulated with DPPE-PEG750 liposome vehicle control, 3M-052 DPPE-PEG750 liposomes, GLA DPPE-PEG750 liposomes, or GLA-3M-052 DPPE-PEG750 liposomes (Fig. 2). While responses varied according to adjuvant dose, overall it was apparent that 3M-052 liposomes induced higher IL-12p70, MCP-1, Mip-1β, and IFN-γ compared to GLA liposomes. The combination of both ligands induced equivalent or additive innate chemokine responses, but induced a synergistic innate Th1 immune response.

### In vivo biological activity

**Fig. 3:**
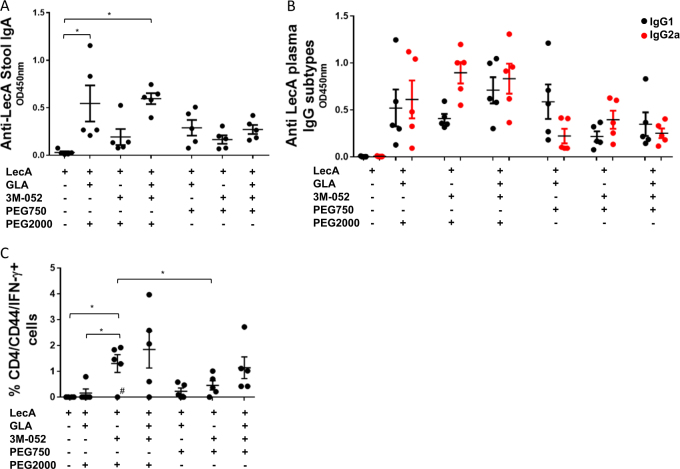
Biological activity of adjuvant formulation: importance of PEG length and complementary roles for GLA and 3M-052. Five mice per group were immunized three times with a 2-week interval between immunizations using a mixed intranasal/subcutaneous regimen. Mice were euthanized a week after third immunization and samples collected. **a** Stool supernatants were diluted 250-fold and anti-LecA IgA titer was determined by ELISA. **b** Plasma samples were diluted 100,000-fold and titers of anti-LecA IgG1 (black circles) and IgG2a (red circles) subtypes were determined by ELISA. **c** Intracellular IFN-γ levels were measured using flow cytometry as described (negative values obtained after subtracting values from unstimulated cells were considered zero; the outlier test described below was conducted prior to this data transformation). Error bars represent standard error of the mean. For the analysis of the data in plot **a**, data were analyzed by one-way ANOVA with Sidak’s correction for selected comparisons. For the analysis of the data in plot **b**, data were log-transformed with a small offset if necessary and Sidak’s correction for selected comparisons was employed. All formulations in plot **b** elicited statistically increased (*p* < 0.05) IgG1 and IgG2a responses compared to antigen alone; for clarity in the figure these statistical bars are not shown. For the analysis of the data in plot **c**, data were analyzed by Welch’s one-way ANOVA with Games–Howell correction for multiple comparisons; two outliers were identified by Grubb’s test (*α* = 0.05) and statistical significance was only achieved when they were excluded from analysis: the low value for the 3M-052 PEG2000 group (indicated by the hash mark in the plot), and a high value (11.8%) in the antigen alone control group (data point not shown in plot)

To determine whether GLA and 3M-052 had complementary adjuvant roles and whether PEG length affected vaccine immunogenicity, we immunized CBA mice using an alternating intranasal/subcutaneous/intranasal regimen as described in our previous publication.^22^ Fecal IgA measured 1 week after the 3rd immunization indicated that GLA-containing formulations elicited the highest IgA responses with no added benefit due to 3M-052 inclusion (Fig. 3a). Conversely, plasma IgG2a titers measured 1 week after the 3rd immunization demonstrated that the highest responses were elicited by formulations containing 3M-052, especially with respect to the IgG2a:IgG1 ratio, with no added benefit of GLA inclusion (Fig. 3b). Likewise, T cell responses analyzed by intracellular cytokine staining indicated that 3M-052-containing formulations drove higher IFN-γ production from CD4 T cells (Fig. 3c). Thus, complementary roles for GLA and 3M-052 were elucidated, motivating inclusion of both TLR ligands in order to elicit a complete profile of mucosal IgA and Th1-type cellular immune responses. The pattern of complementary roles for GLA and 3M-052 was evident regardless of the PEG length that was employed in the formulation. However, an overall increase in magnitude of response was apparent due to inclusion of PEG2000 compared to PEG750 (Fig. 3a–c). Subsequent studies employed the GLA-3M-052-PEG2000 composition.

### Effect of immunization route on response profile

**Fig. 4:**
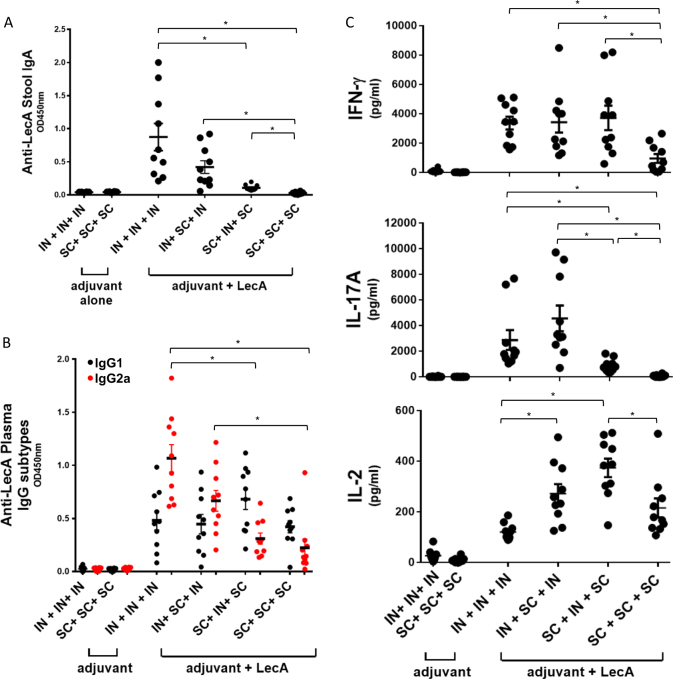
Effect of immunization route on response. Ten mice per group were immunized three times with a 2-week interval between immunizations via intranasal (IN) or subcutaneous (SC) route or a combination of these using GLA-3M-052-PEG2000 liposomes as an adjuvant. Samples were collected 1 week after 3rd immunization. **a** Stool supernatants were diluted 400-fold to determine LecA-specific IgA titer by ELISA. **b** Plasma samples were diluted 100,000-fold to determine titers of IgG1 (black circles) and IgG2a (red circles) subtypes. **c** Splenocytes were restimulated with LecA for 72 h and production of extracellular cytokines in the culture supernatants was determined by Luminex. Cytokine levels of the unstimulated samples were at the baseline (not shown). Error bars represent standard error of the mean. For clarity, statistical significance vs. the adjuvant alone control groups is not represented but is detailed below. For the analysis of the data in plot **a**, Welch’s one-way ANOVA was employed with Games–Howell correction for multiple comparisons; all of the vaccine groups except for SC + SC + SC were statistically different (*p* < 0.05) from the adjuvant alone groups. For the analysis of the data in plot **b**, data were log-transformed and one-way ANOVA with Tukey’s correction for multiple comparisons was employed; all of the vaccine groups were statistically different (*p* < 0.05) from the adjuvant alone groups for both IgG2a and IgG1, and statistically significant differences between vaccine groups represent IgG2a only since no statistical differences were found between vaccine groups for IgG1. For the analysis of the data in plot **c**, data were log-transformed with a small offset as necessary and Welch’s ANOVA with Games–Howell correction for multiple comparisons was employed (outliers were maintained since they did not affect statistical significance); all vaccine groups were statistically different (*p* < 0.05) from the adjuvant alone groups for IFN-γ production, and the adjuvant alone IN + IN + IN was statistically different from the adjuvant alone SC + SC + SC; for IL-17A production, all vaccine groups except for SC + SC + SC were significantly different (*p* < 0.05) from the adjuvant alone groups; for IL-2 production, all vaccine groups were significantly different (*p* < 0.05) from the adjuvant alone groups

In order to promote generation of both mucosal and systemic immune responses we employed an alternating intranasal and subcutaneous immunization regimen in our previous study.^22^ To more fully assess the impact of immunization regimen in the present work, we evaluated fecal IgA, plasma IgG2a:IgG1, and cytokine (IFN-γ, IL-17A, and IL-2) production from LecA-stimulated splenocytes in mice immunized three times subcutaneously, intranasally, or alternating combinations of both routes. As expected, the intranasal-only regimen resulted in the highest fecal IgA levels (Fig. 4a) with little response evident in the mice immunized by the subcutaneous-only regimen. Surprisingly, the intranasal-only regimen also resulted in the highest IgG2a response (Fig. 4b) coupled with significant LecA-specific cytokine production from splenocytes (Fig. 4c). While IFN-γ levels were highest and comparable between the regimens containing at least one dose of intranasal immunization, IL-17A production was favored by intranasal priming (Fig. 4c). Interestingly, subcutaneously primed mixed regimen showed significantly more IL-2 production compared to subcutaneous-only or intranasal-only regimens. Other than IL-2 production, no significant benefit was apparent in the subcutaneous regimen or the more complicated alternating route regimens compared to the intranasal-only regimen. Moreover, the significance of IL-2 in protection from amebiasis in humans or the mouse model is not known. Overall, LecA + GLA-3M-052-PEG2000 liposomes administered intranasally resulted in a complete immune response profile considered necessary for protective efficacy in humans and/or mice, including mucosal IgA and Th1/Th17 cellular immunity.

### Protection studies

**Fig. 5:**
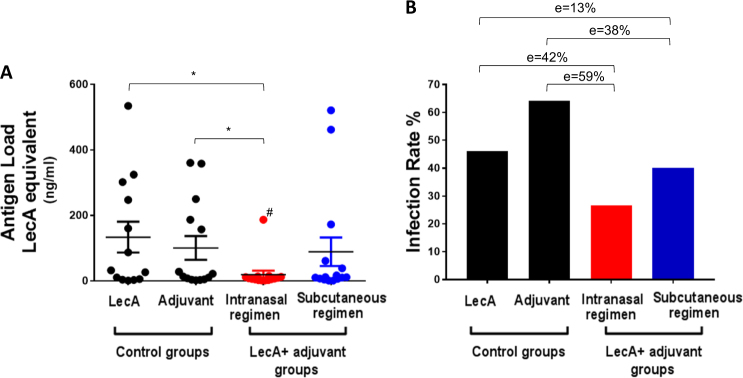
Intranasal immunization protected from intestinal amebiasis in a mouse model. Mice were immunized three times using an intranasal or a subcutaneous regimen with a 2-week interval between immunizations. Mice were challenged intracecally with a virulent strain of *E. histolytica* 4 weeks past 3rd immunization. Mice were euthanized a week after challenge and cecal contents analyzed for **a** antigen load using ELISA and **b** live amebae by culture as a measure of sterile protection. Group sample size was 13–15 mice as specified in the Materials and Methods section. Both LecA-only and adjuvant-only control groups received intranasal immunization. Antigen load in plot **a** was log-transformed to generate normal distributions, and one data point was identified as an outlier based on Grubb’s test (the data point is indicated by the hash mark in the plot); the statistical significance (*p* < 0.05) of the intranasal adjuvanted vaccine group vs. the controls was performed using Welch’s ANOVA with Games–Howell’s correction for multiple comparisons. Statistical significance was only achieved if the outlier data point was not included in the statistical analysis. Error bars represent standard error of the mean. The efficacies (*e*) in plot **b** were calculated with regard to the control groups, although statistical significance (*p* < 0.05) was not achieved for any group after the Bonferroni multiple comparison correction was performed

The protective efficacy of LecA + GLA-3M-052-PEG2000 liposomes was evaluated in the mouse model of amebic colitis developed by our lab.^25^ Mice were immunized three times intranasally or subcutaneously and subsequently challenged intracecally with *E. histolytica* 4 weeks following the last immunization. Mice were euthanized a week after challenge and cecal contents analyzed for antigen load by ELISA as well as for the presence of live ameba by culturing in growth medium. Antigen load was reduced by over 80% (or 90% when outlier data point is excluded) in mice immunized intranasally compared to control groups as determined from the mean cecal LecA values (Fig. 5a). Subcutaneous immunization, in contrast, was not effective in reducing antigen load. Furthermore, infection rate in intranasally immunized mice was reduced by 59% compared to animals receiving adjuvant alone, and by 42% compared to animals receiving antigen alone (Fig. 5b). Subcutaneous route on the other hand reduced the infection rate by 33 and 13% in comparison with adjuvant or LecA-only control group, respectively. Statistical significance (*p* < 0.05) was not achieved in the infection rate group comparisons (Fig. 5b) after the analysis was corrected for multiple comparisons, whereas antigen load reduction was statistically significant when the outlier data point (indicated by the hash mark in Fig. 5a) was removed.

## Discussion

The work described here was built on our previously published studies to develop a novel protective recombinant adjuvanted vaccine for amebiasis. Important advancements described in the present studies include demonstrating unique and complementary roles for the two different TLR ligands employed (GLA and 3M-052), optimization of the adjuvant formulation composition (replacing PEG750 with PEG2000) for enhanced immunogenicity, and simplifying the immunization regimen to three intranasal administrations while maintaining mucosal antibody and systemic Th1/Th17 immune responses. Together, these improvements resulted in enhanced protective efficacy in the amebic colitis mouse model. Greater than 80% reduction in antigen load demonstrated here for intranasally administered LecA + GLA-3M-052-PEG2000 liposomes appears to be improved over 46% seen for LecA + GLA-3M-052-PEG750 liposomes administered using a combination administration regimen reported previously.^22^ Moreover, an efficacy of 59% based on culture data shown by the present adjuvanted vaccine appears to be an improvement over the 34% reported previously.^22^ While caution should be used in comparing different studies performed at different times, this apparent improvement in efficacy is likely due to the optimization of liposome composition and immunization route.

Recent work has indicated promising potential for TLR4/TLR7-8 adjuvant formulations.^19,26–28^ TLR7-8 ligands have particular relevance for vaccines designed for infants.^29^ Nevertheless, the increased complexity of such an approach requires demonstration of added benefit of each TLR ligand to justify further development. It was previously reported^30,31^ that intranasal administration of an aqueous nanosuspension of GLA in mice increased mucosal IgA and serum IgG titers, and Th1 and/or Th17 type cellular responses, consistent with our results. Interestingly, in minipigs, intranasal administration of a TLR7/8 ligand (R848) in combination with an aqueous nanosuspension of GLA resulted in reduced serum IgG and IgA responses compared to R848 alone.^32^ In contrast, we found that GLA and 3M-052 demonstrated complementary roles, with GLA favoring production of mucosal IgA, whereas 3M-052 drove plasma IgG2a and CD4 T cell IFN-γ responses. Such discrepancies could be attributable to significant differences in formulation compositions, the TLR7/8 ligand structure (R848 vs. 3M-052), or the animal model. In our hands, while intranasal immunization with adjuvanted amebiasis vaccine cleared more than 80% cecal antigen upon challenge, subcutaneous immunization failed to clear antigen. Thus, present data indicates a superior protection capability of the optimized intranasal formulation. We note here that a potential limitation in interpretation of the data is the lack of antigen alone or adjuvant alone controls for the subcutaneous-only regimen. Also, *E. histolytica* cystic form, the infective stage, has not been detected in mice. This limits our ability to assess the effect of vaccination on transmission using this model.

The ability of the GLA-3M-052 liposome adjuvant to elicit mucosal IgA and systemic Th1/Th17-type immune responses is important since these readouts are associated with protection from amebiasis. Moreover, vaccines against other enteric diseases such as cryptosporidiosis may require a similar immune response profile for protective efficacy.^33^ Evaluation of the GLA-3M-052 liposome adjuvant with other vaccine antigens and disease models will be required to determine suitability for other enteric diseases. The potential practical benefits of an intranasally administered vaccine are many, including needle-free delivery, ability to administer without specialized medical training, and enabling of both mucosal and systemic immunity.^34^ Potential challenges of intranasal administration in a global health setting include the difficulty in preparing multidose vials without risking contamination between users, and overcoming safety concerns stemming from an intranasal influenza vaccine product previously licensed in Europe that was associated with an elevated risk of Bell’s palsy (potentially attributable to the *E. coli* heat-labile toxin adjuvant component).^35^ In this context, it is important to highlight that the TLR ligands described in the present work are synthetic and highly pure molecules. Mice did not show any visible side effects upon immunization in terms of local reaction or behavior during the course of experiments, although comprehensive toxicity studies have not yet been performed.

Regarding the effects of PEG length, PEG750 liposomes appeared to be more physically stable under accelerated conditions than PEG2000 liposomes, and PEG2000 liposomes appeared to be more susceptible to particle size change upon mixing with antigen. Although both liposome compositions demonstrated acceptable overall stability, the increased physical stability of the PEG750 liposomes compared to PEG2000 liposomes is somewhat unexpected since longer PEG length is expected to provide greater steric stability to liposomes; therefore, the mechanism for greater stability in the PEG750 liposomes is unclear. Nevertheless, overall increased LecA-specific immune responses were evident with PEG2000 liposomes compared to PEG750 liposomes. There is general agreement in the literature that PEGylation, including PEG length and density, is correlated with reduced non-specific protein adsorption on liposomes and other nanoparticles (the “protein corona” effect), resulting in increased circulation times in the case of intravenous drug delivery or faster drainage from the injection site to the lymph node in the case of intramuscular, subcutaneous, or intranasal immunization. In particular, PEG2000 appears to be more effective in this regard than shorter PEG lengths.^36,37^ Interestingly, Pozzi et al. reported that PEG2000 cationic liposomes showed less non-specific protein bound to the liposome surface upon exposure to human plasma compared to liposomes decorated with PEG1000; moreover, a higher proportion of the proteins bound to the PEG2000 liposomes was comprised of opsonization-promoting classes of proteins such as IgG and complement,^37^ which are expected to enhance lymph node retention.^38^ Following this line of reasoning, it is possible that the PEG2000 liposomes in the present work allow more effective lymph node delivery and retention following immunization due to the altered protein corona composition. Further investigation along these lines is merited; for instance, delivery and uptake of PEG750 or PEG2000 liposomes is likely dependent on the density of the PEG layer.^39^

Finally, the tissue-specific expression patterns of TLRs and the complex interplay between different innate pathways require further study to elucidate the mechanisms underlying the complementary roles of intranasally administered TLR4 and TLR7/8 ligands demonstrated here.^32^ For instance, five subsets of antigen presenting cells have been recently identified within the mouse nasal tissue, the majority of which appear to be of the CD11b^+^ phenotype.^40^ These cells express TLR4 in some mouse strains, whereas there is conflicting evidence regarding whether this phenotype expresses TLR7 (TLR8 is refractory in the mouse model).^41^ Increased TLR4 expression in nasal tissue could explain why intranasally administered TLR4 ligands such as GLA enhance mucosal IgA. Compared to GLA, the TLR7/8 ligand 3M-052 elicited a stronger Th1-type response in the present work, which is consistent with previous results from our lab where an aluminum salt formulation of 3M-052 induced higher levels of antigen-specific IFN-γ, TNF, IL-2, and serum IgG2 compared to the same formulation with GLA.^42^ Plasmacytoid dendritic cells produce high levels of type 1 interferons in response to stimulation of TLR7 but do not express TLR4,^41,43,44^ which could help explain the potent Th1-inducing activity of 3M-052 in the present work. Thus, GLA and 3M-052 may be activating distinct subsets of dendritic cells, resulting in complementary adjuvant activity. Indeed, one report found that TLR7 and TLR4 ligands stimulated different levels of IL-12 from mouse splenic dendritic cells depending on the cell phenotype.^45^ The production of cytokines and chemokines from human whole blood stimulated in vitro by the adjuvant formulations is consistent with the Th1-biasing profile of 3M-052 liposomes evident in the in vivo studies.

We note that the present work did not evaluate the effect of liposomes in the absence of TLR ligands in the in vivo studies; nevertheless, the in vitro assay demonstrated no stimulatory activity which is consistent with our previous work on PEGylated liposomes with a recombinant influenza antigen indicating minimal effects on Th1-type immune responses in mice by the liposomal vehicle in the absence of TLR ligands.^46^ Additional questions remaining to be investigated include longevity of immune response elicited and how it compares to previous reports of GLA-based adjuvant formulations.^47^

The present work represents a significant step forward from our previously published results on dual TLR ligand liposome-adjuvanted LecA by identifying complementary roles for GLA and 3M-052, demonstrating enhancement of adjuvant potency by modulation of liposome PEG length, and maintaining mucosal antibody and systemic Th1/Th17-type immune responses with a simplified mucosal immunization regimen. The refined liposome composition enhanced LecA-specific fecal IgA and Th1/Th17 cellular immunity, resulting in 59% protective efficacy against *E. histolytica* in mice immunized intranasally. GLA-3M-052-PEG2000 is a promising clinical adjuvant candidate since it is based on completely synthetic and biocompatible components that have been produced under cGMP conditions and are in clinical testing with other products. Thus, LecA + GLA-3M-052-PEG2000 represents an exciting vaccine candidate meriting further development in preparation for potential clinical testing as a first-in-human amebiasis vaccine candidate.

## Methods

### Preparation of LecA antigen and adjuvant formulation

LecA antigen was manufactured by TECHLAB, Inc. (Blacksburg, VA) as described^16^ and stored in phosphate buffered saline at 5 °C. Glucopyranosyl lipid adjuvant (GLA), 1,2-dipalmitoyl-sn-glycero-3-phosphocholine (DPPC), 1,2-distearoyl-sn-glycero-3-phosphoethanolamine-N-[methoxy(polyethylene glycol)-750] (DSPE-PEG750), and 1,2-distearoyl-sn-glycero-3-phosphoethanolamine-N-[methoxy(polyethylene glycol)-2000] (DSPE-PEG2000) were obtained from Corden Pharma (Liestal, Switzerland), Avanti Polar Lipids (Alabaster, AL), or Lipoid LLC (Newark, NJ). 3M-052 was provided courtesy of 3M Drug Delivery Systems (St. Paul, MN). Cholesterol and buffer salts were purchased from J.T. Baker (San Francisco, CA) or Sigma (St. Louis, MO).

PEGylated liposome formulations were manufactured by combining DPPC, cholesterol, DSPE-PEG750 or DPPE-PEG2000, and 3M-052 and/or GLA in chloroform. The lipid molar ratio was 9.8:5.7:0.8 (DPPC:cholesterol:PEGylated lipid). The organic solvent from the formulations was then evaporated for at least 12 h using a rotary evaporator. The lipid film was rehydrated in 25 mM ammonium phosphate buffer (pH ~5.7) and sonicated in a Crest Powersonic CP230D (Trenton, NJ) water bath at ~60 °C for up to 2 h until the lipid film had separated from the sides of the glass flask. The formulation was then microfluidized at 30,000 psi for ~5 passes with a recirculating water chiller set at 10 °C. Liposomes were filtered through a 0.8/0.2 µm double membrane polyethersulfone filter and stored at 5 and 37 °C. Smaller batches of liposomes employed in the in vitro human whole blood assay were made with DPPE-PEG750 instead of DSPE-PEG750, hydrated with phosphate-buffered saline instead of ammonium phosphate buffer, and were manufactured by sonication only (without microfluidization). It should be noted that liposomes intended for subcutaneous administration were manufactured to contain lower concentrations of GLA and 3M-052 compared to liposomes intended for intranasal administration due to administration volume considerations (100 vs. 20 µL, respectively), whereas phospholipid concentration was constant regardless of administration route. Thus, liposomes intended for subcutaneous administration were manufactured at 7.2 mg/ml DPPC, 1.2 mg/ml DSPE-PEG750 or 2.2 mg/ml DSPE-PEG2000, 2.2 mg/ml cholesterol, 0.1 mg/ml GLA, and 0.04 mg/ml 3M-052; liposomes intended for intranasal administration were manufactured at these same phospholipid and cholesterol concentrations with 0.5 mg/ml GLA and 0.2 mg/ml 3M-052. All liposomes were mixed 1:1 by volume with LecA/saline prior to immunization.

### Physicochemical characterization of adjuvant formulation

The concentrations of GLA and 3M-052 were determined by reverse-phase HPLC with charged aerosol detection. The HPLC method was essentially as described previously.^48^ For 12–16 month timepoints of 3M-052-containing formulations, the concentration of 3M-052 was determined by reverse-phase HPLC with UV detection (321 nm); the HPLC method in this case consisted of first diluting the formulation 10-fold in isopropanol containing 0.5% trifluoroacetic acid and then eluting the sample on a Zorbax Bonus RP column (4.6 × 150 mm, 3.5 µm) at 45 °C with a 1 ml/min flow rate while employing a gradient mobile phase consisting of Mobile Phase A (0.1% trifluoroacetic acid in water), Mobile Phase B (methanol), and Mobile Phase C (isopropanol) as follows: initial (85% A, 15% B), 2.5 min (60% A, 40% B), 17.5 min (5% A, 40% B, 55% C), 22.0 min (5% A, 40% B, 55% C), 22.5 min (85% A, 15% B). Liposome particle size and size polydispersity were evaluated using a Malvern Instruments (Worcestershire, UK) Zetasizer Nano-S or Nano-ZS. The formulation was diluted 10-fold or 100-fold in ultrapure (18.2 MΩ) water in a 1.5 ml polystyrene disposable cuvette. In general, three separate cuvettes were prepared for each sample and size measurements were then made three times for each cuvette. The data shown represent a single batch of formulation with the indicated composition; multiple batches of each composition were not manufactured.

### Physicochemical compatibility of antigen and adjuvant after mixing

The short-term (≤24 h) physicochemical compatibility of the antigen-adjuvant mixture was evaluated by monitoring particle size, visual appearance, and antigen primary structure immediately after mixing and 4 and 24 h after mixing, with mixtures stored at 5 °C and ambient temperature. The antigen was first diluted in saline to 0.1 mg/ml, and subsequently mixed in 1:1 volume with the liposomal adjuvant formulation. Particle size was measured as described above except that one cuvette was prepared instead of three. SDS-PAGE was conducted by mixing 50 µL sample with 50 µL 4× reducing sample buffer and 100 µL 20% SDS. The sample was heated at 90 °C for 5 min and stored at −20 °C, after which it was reheated for 1 min at 90 °C and loaded onto a polyacrylamide gel with Tris-glycine running buffer for 65 m at 180 V, followed by staining with Coomassie blue. Gels were processed in parallel and derive from the same experiment, which was performed once according to a well-established protocol performed regularly in our lab.

### Human whole blood assay

Heparinized human blood samples were collected from three normal, healthy male donors using standard phlebotomy techniques. The sample size of three donors was not powered to detect statistical differences but rather to represent general trends. This experiment was performed once using a well-established assay regularly employed in our lab. All formulations were serially diluted in two-fold steps in irrigation-grade saline, then 20 μl of each serial dilution was added to 96-well tissue-culture grade U-bottom plates, followed by the addition of 180 μl of whole blood, providing a final well volume of 200 μl with a top formulation concentration of 4 μg/ml GLA and/or 1.5 µg/ml 3M-052. Duplicate wells were prepared for each donor. Samples were incubated for 24 h at 37 °C/5% CO_2_ in a humidified incubator and plasma supernatants assayed by ELISA for the selected cytokines (Mip-1β [R&D Systems, catalog #DY271]; IL-12p70 [eBioscience, catalog #88-7126-86]; IFN-γ [eBioscience, catalog #88-7316-86]; MCP-1 [eBioscience, catalog #88-7399-88]).

### Immunizations

Four to 6-week-old male CBA/J mice (Jackson Labs) were used for all the experiments. Typically 5 μg of LecA was mixed with liposome formulations containing appropriate TLR ligand/s (5 μg GLA; 1–2 μg 3M-052) prior to immunizations. The volume was brought up with saline to 100 μl for subcutaneous or to 20 μl for intranasal immunization. Subcutaneous injections were given in the neck region, whereas intranasal immunizations were carried out under anesthesia and 10 μl of antigen-adjuvant mix was administered per nostril. A 2-week interval was maintained between the consecutive immunizations for all the regimens, which consisted of three immunizations total. For immunogenicity experiments, 5 mice per group were employed for the data shown in Fig. 3, whereas 10 mice per group were used for the data shown in Fig. 4; for the protective efficacy experiment, 13–15 mice per group were analyzed (13 for the antigen-only control group, 14 for the adjuvant-only control group, and 15 for the adjuvanted vaccine groups). Each mouse experiment was performed once. Sample size was determined based on our previous experience evaluating adjuvant formulations in this model with the aim of using a minimum number of animals possible to generate meaningful results in immunogenicity and efficacy. Animal experiments were not randomized.

### Measurement of immunogenicity

Mice were euthanized a week after final immunization for immunogenicity experiments (stool IgA, plasma IgG, cytokines). Antibody titers were measured by ELISA using 96-well plates coated with 0.5 μg LecA per well. Horseradish peroxidase-conjugated secondary antibodies specific for IgA (catalog #1040-05) and IgG (catalog #1071-05 and 1081-05) subtypes were purchased from Southern Biotechnology and diluted as per the manufacturer’s instructions. Antibody units were calculated using standard curves. For the measurement of extracellular cytokines, 250,000 splenocytes were re-stimulated with 50 μg/ml LecA in 200 μl RPMI1640 complete medium (supplemented with 10% heat-activated fetal bovine serum (Gemini Labs), 2.05 mM L-Glutamine, 10,000 U/ml Penicillin, and 10 mg/ml Streptomycin) for 72 h and undiluted supernatant analyzed by a multiplex suspension array system using Luminex beads (Bio-Plex 200, Bio-Rad). For intracellular cytokine staining, 1 × 10^6^ splenocytes were cultured in complete RPMI medium and re-stimulated with 10 μg/ml LecA for 22 h. Anti-CD28 and Golgiplug (BD Biosciences) were added for the last 12 h of stimulation, followed by surface staining with anti CD4 PerCp-Cy5.5 (BioLegend, catalog #100433) and anti CD44 APC-Cy7 (BioLegend, catalog #103027). Cells were permeabilized and stained with anti IFN-γ PE-Cy7 (BioLegend, catalog #505825) and run on a BD FACSCalibur (BD Biosciences). The data were analyzed with FlowJo software (Fig. S5). All antibodies were purchased from BD Biosciences.

### Culture conditions and challenge experiments

Virulent trophozoites, originally derived from HM1:IMSS (ATCC) and passed sequentially through mice were maintained in a trypsin-yeast extract-iron (TYI-S-33) medium supplemented with 2% Diamond vitamins, 13% heat-inactivated bovine serum and 100 U/ml penicillin plus 100 μg/ml streptomycin (Invitrogen). Mice from immunized and control groups were challenged intracecally 4 weeks after the final boost with 2 million trophozoites in 150 μl medium following laparotomy. Mice were euthanized a week after the challenge. Cecal contents were suspended in 1 ml PBS, 300 μl were cultured anaerobically in TYI-S-33 broth at 37 ^°^C for 5 days and 200 μl used for antigen load ELISA. Culture results were scored in a blinded manner. Vaccine efficacy was calculated as 100 × 1−(% of vaccinated mice with infection)/(% of sham mice with infection).

### Fecal antigen detection

Fecal antigen in the cecal contents was detected using the *E. HISTOLYTICA* II ELISA kit (TechLab Inc., catalog #T5017). An optical density at 450 nm of ≥0.05 above the negative control was considered positive. A standard curve was generated using purified LecA.

### Statistical analysis

Statistical analyses were performed using Graph Pad Prism software and Microsoft Excel (www.biostathandbook.com/welchanova.xls). While not pre-established prior to the experiments, the statistical criteria below were followed consistently, and take into account normality, similarity of variance, outliers, and corrections for multiple comparisons. Proportions of infected and uninfected mice from challenge trials were analyzed using one-sided Fisher’s exact test with Bonferroni’s correction for multiple comparisons. Antigen load, antibody titers, and cytokine levels were analyzed by first testing for normality using the D’Agostino & Pearson normality test (*α* = 0.05). If all groups passed the normality test, they were then tested for similarity of variance using the Brown–Forsythe test (*p* < 0.05). If variances between groups was similar, they were analyzed using one-way ANOVA with Tukey’s or Sidak’s correction for multiple comparisons as indicated. If data did not pass the normality test, the data were log-transformed and re-tested for normality. If the normality test was still not passed, Grubb’s test for outliers (*α* = 0.05) was employed to remove outliers. If original or log-transformed data did not pass the similarity of variances test, the data were analyzed by Welch’s ANOVA with Games–Howell multiple comparison correction. In cases where small sample size did not allow for testing of normality by the D’Agostino & Pearson method, the data were not transformed or were treated according to the method found most appropriate for the same assay when larger sample sizes were available. Prior to log-transformation of data sets containing zero values, the values were offset by the smallest value present in the rest of the data set. *p*-values of less than 0.05 were considered statistically significant. Center values are represented as means, and error bars represent standard deviation or standard error of the mean as indicated. See figure captions for more specific details regarding the statistical analysis of each set of data.

### Ethics statement

All animal studies were conducted in strict accordance with the Guide for the Care and Use of Laboratory Animals (8th edition) of the National Institutes of Health. The protocol was approved by the International Animal Care and Use Committee at the University of Virginia (Protocol #4126; PHS Assurance #A3245-01). All surgeries were performed under ketamine/xylazine anesthesia; analgesics and supportive care was given to facilitate the well-being of the research animals. The human subjects’ involvement in routine blood draws was approved by Western Institutional Review Board (WIRB) and each research participant signed a WIRB-approved informed consent form.

### Data availability

The data that support the findings of this study are available from the corresponding author upon reasonable request.

## Electronic supplementary material

Supplementary Information
