# Supplementary material for: Adjuvant composition and delivery route shape immune response quality and protective efficacy of a recombinant vaccine for Entamoeba histolytica
Source: NPJ Vaccines. 2018 Jun 5;3:22. doi: 10.1038/s41541-018-0060-x (PMC5988657; doi:10.1038/s41541-018-0060-x)
Supplement: Supplementary file 1 — Supplementary Information [file 41541_2018_60_MOESM1_ESM.pdf]

Supplementary Information for Abhyankar et al. "Adjuvant Composition and Delivery Route Shape Immune Response Quality and Protective Efficacy of a Recombinant Vaccine for *Entamoeba Histolytica*"

This supplementary information contains additional physicochemical stability data of liposomes manufactured with various concentrations of TLR ligands (Figures S1-S3). Also included is physicochemical compatibility data of the adjuvant formulations with the recombinant LecA vaccine antigen (Figure S4) and representative flow cytometry gating strategy (Figure S5).

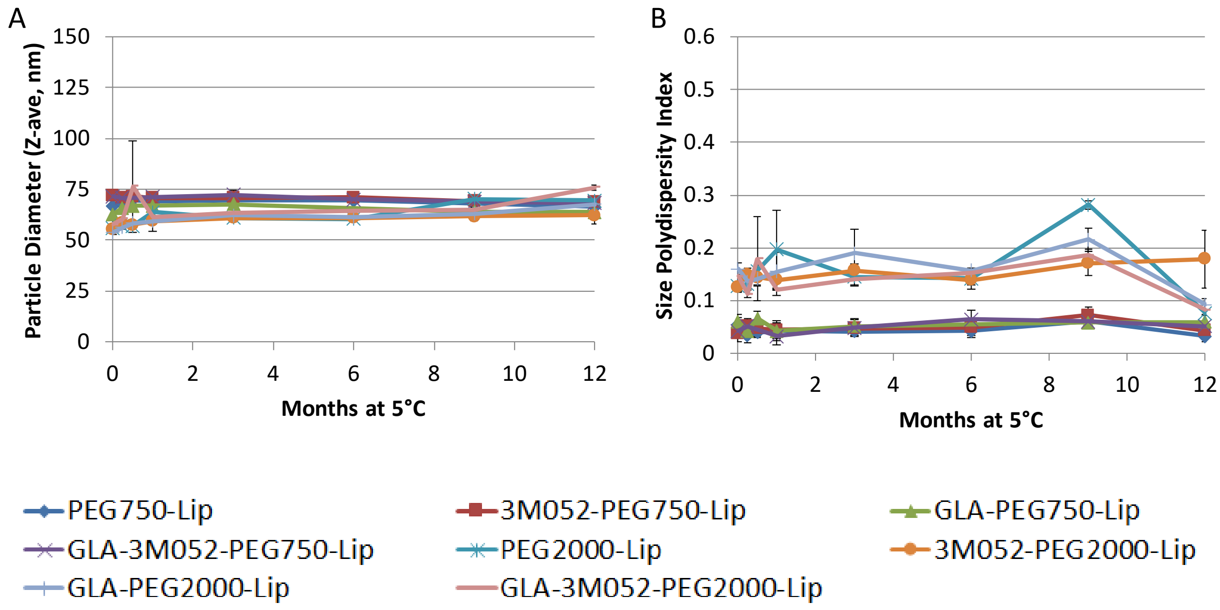

Figure S1. Effect of PEG length and adjuvant composition on liposome physical stability. (A) Particle size and (B) size polydispersity of PEGylated liposomes with indicated compositions and stored at 5°C. Error bars represent the standard deviation from 9 measurements (3 measurements from each of 3 cuvettes).

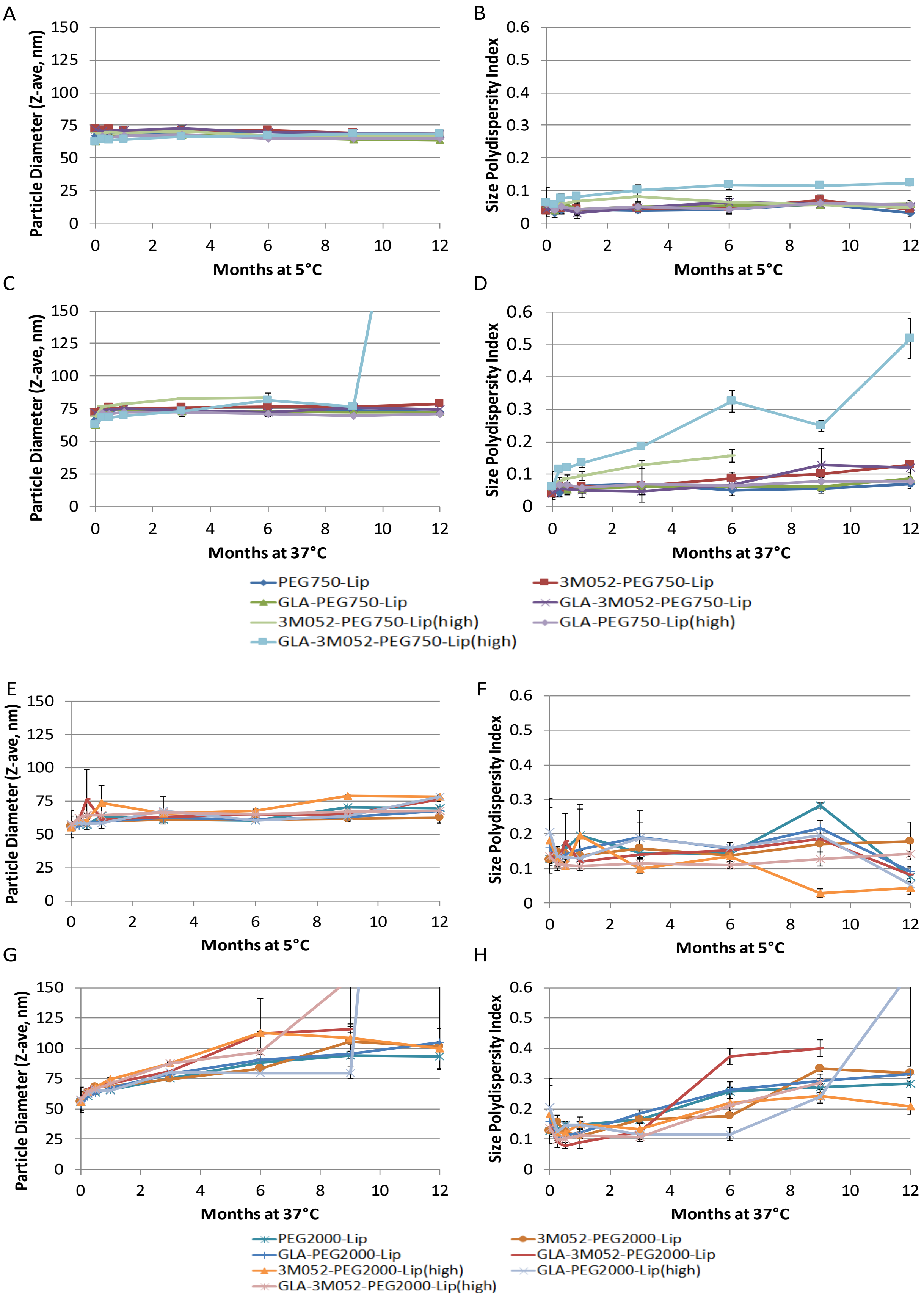

A
Particle Diameter (Z-ave, nm)
Months at 5°C
B
Size Polydispersity Index
Months at 5°C
C
Particle Diameter (Z-ave, nm)
Months at 37°C
D
Size Polydispersity Index
Months at 37°C
PEG750-Lip
3M052-PEG750-Lip
GLA-PEG750-Lip
GLA-3M052-PEG750-Lip
3M052-PEG750-Lip(high)
GLA-PEG750-Lip(high)
GLA-3M052-PEG750-Lip(high)
E
Particle Diameter (Z-ave, nm)
Months at 5°C
F
Size Polydispersity Index
Months at 5°C
G
Particle Diameter (Z-ave, nm)
Months at 37°C
H
Size Polydispersity Index
Months at 37°C
PEG2000-Lip
3M052-PEG2000-Lip
GLA-PEG2000-Lip
GLA-3M052-PEG2000-Lip
3M052-PEG2000-Lip(high)
GLA-PEG2000-Lip(high)
GLA-3M052-PEG2000-Lip(high)

Figure S2 (preceding page). Effect of adjuvant concentration (GLA and 3M-052) on liposome physical stability. Target GLA concentration was either 0.1 mg/ml or 0.5 mg/ml (high). Target 3M-052 concentration was either 0.04 mg/ml or 0.2 mg/ml (high). (A) Particle size and (B) size polydispersity of PEG750 liposomes stored at 5°C. (C) Particle size and (D) size polydispersity of PEG750 liposomes stored at 37°C. (E) Particle size and (F) size polydispersity of PEG2000 liposomes stored at 37°C. (G) Particle size and (H) size polydispersity of PEG2000 liposomes stored at 37°C. In some cases at the 37°C storage conditions, formulations failed visual stability testing (due to visual inhomogeneities) and were not measured for particle size at 9 or 12 months.

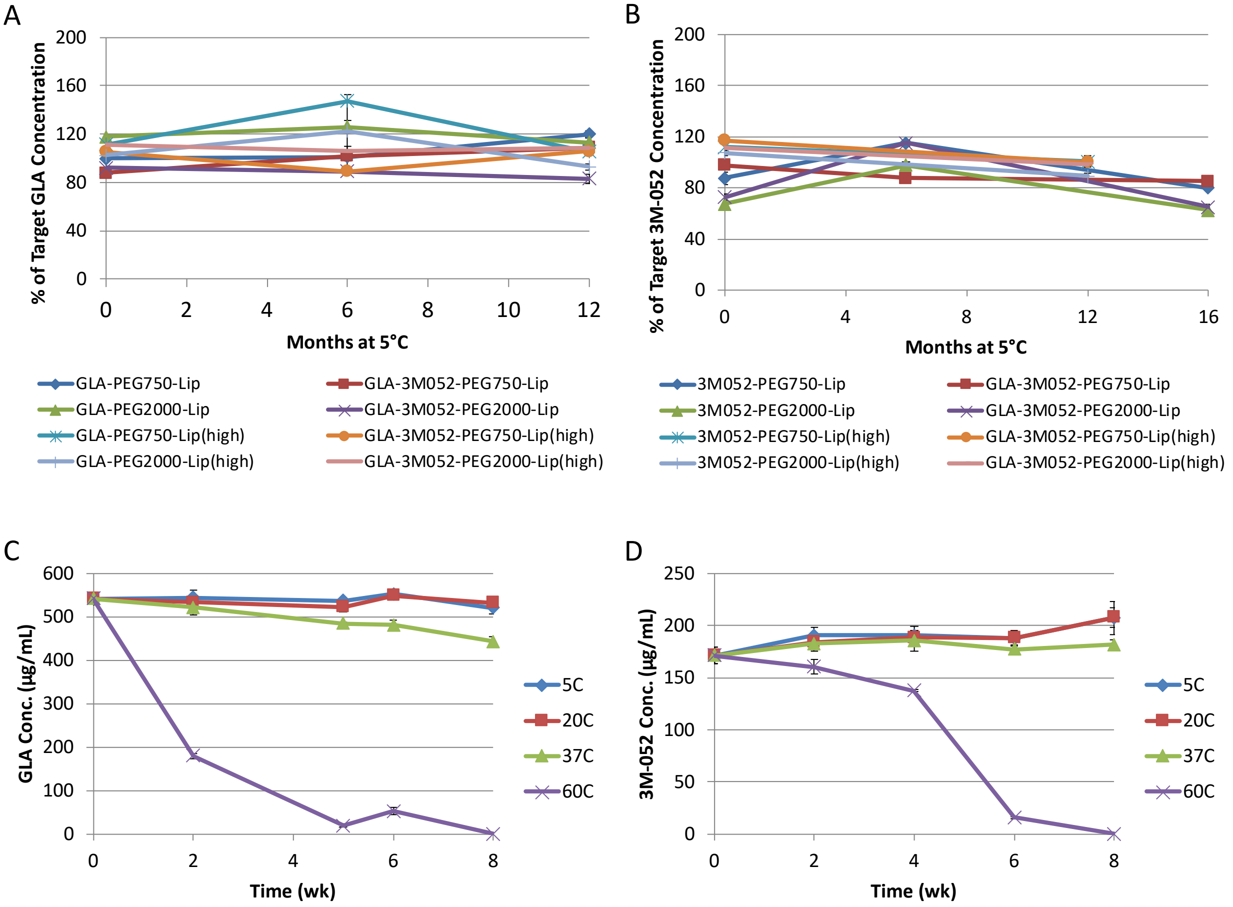

Figure S3. Liposomal GLA and 3M-052 are chemically stable for at least 12 months at 5°C. (A) Percent change in GLA concentration over time in various liposome formulations as monitored by HPLC-CAD. Target GLA concentration was either 0.1 mg/ml or 0.5 mg/ml (high). (B) Percent change in 3M-052 concentration over time in various liposome formulations as monitored by HPLC-CAD or HPLC-UV methods. Target 3M-052 concentration was either 0.04 mg/ml or 0.2 mg/ml (high). (C) Effect of storage temperature on GLA concentration in a selected batch of GLA-3M052-PEG2000-Liposomes. Note that time zero measurement for GLA content was actually collected one month previous to the start of the temperature study during which samples were stored at 5°C. (D) Effect of storage temperature on 3M-052 concentration in a selected batch of GLA-3M052-PEG2000 Liposomes. For all panels, error bars represent standard deviations of three measurements.

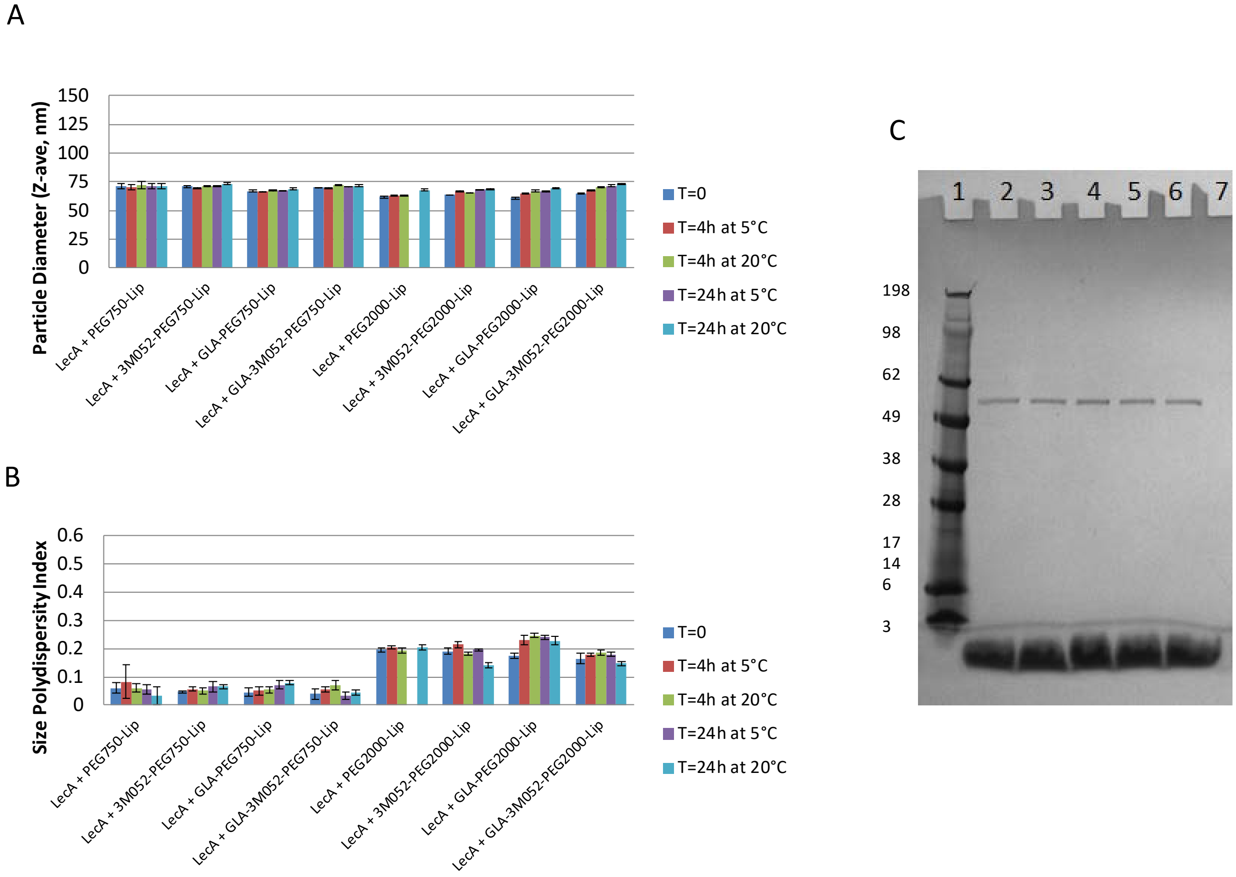

Figure S4. Mixtures of GLA-3M052-Liposomes and LecA antigen are stable for at least 24 h. (A) Particle size of adjuvant formulations after mixing with LecA antigen and saline diluent. (B) Size polydispersity values of adjuvant formulations after mixing with LecA antigen and saline diluent. (C) Representative SDS-PAGE profile of LecA antigen after mixing with adjuvant formulation (GLA-PEG750 Liposomes): Lane 2 T=0, Lane 3 T=4h 5°C, Lane 4 T=4h 25°C, Lane 5 T=24h 5°C, Lane 6 T=24h 25°C. Other adjuvant formulations resulted in similar SDS-PAGE results. For panels A and B, error bars represent standard deviation of three measurements.

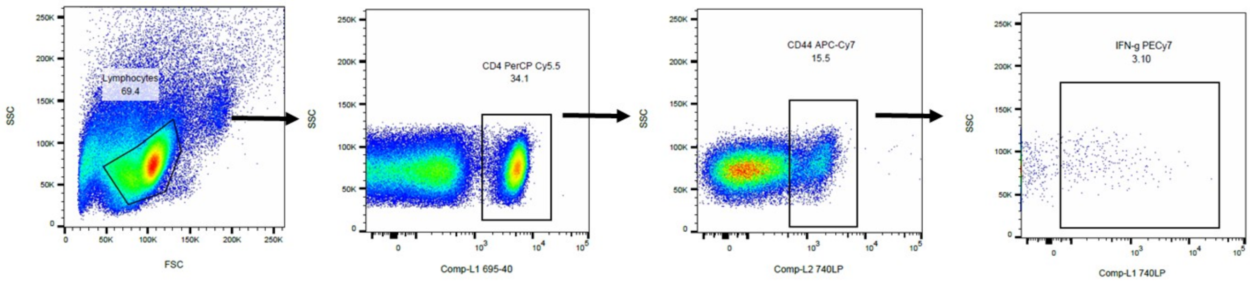

Figure S5. Representative figure showing gating strategy used in flow cytometry analysis to detect $CD4^{+}$ $CD44^{+}$ IFN-g^{+} T-cells.
